# Factors influencing the quality of life of children with cochlear implants^☆^^☆☆^

**DOI:** 10.1016/j.bjorl.2019.01.004

**Published:** 2019-02-22

**Authors:** Joice de Moura Silva, Midori Otake Yamada, Elida Garbo Guedes, Adriane Lima Mortari Moret

**Affiliations:** a Universidade de São Paulo (USP), Faculdade de Odontologia (FOB), Bauru, SP, Brazil; b Universidade de São Paulo (USP), Hospital de Reabilitação de Anomalias Craniofaciais da Universidade (HRAC), Bauru, SP, Brazil

**Keywords:** Quality of life, Child, Cochlear implant, Qualidade de vida, Criança, Implante coclear

## Abstract

**Introduction:**

The multidimensional impact of hearing loss on the various demands of life in children using cochlear implants is represented by variables that can influence the hearing, language and quality of life outcomes of this population.

**Objective:**

To evaluate the factors influencing the quality of life of children with cochlear implantation, considering age, hearing age, age at evaluation, hearing skills, spoken language, family degree of receptiveness, schooling and socioeconomic status of the parents.

**Methods:**

Participated 30 children using cochlear implants, aged 6 to 12 years and their respective parents. The children were evaluated by the categories auditory performance, by language category, and by the children with cochlear implants: perspectives parents questionnaire. Parents were assessed by the family involvement scale.

**Results:**

The cochlear implant impacted the quality of life of the children, with more significant results on the increase of the social relations domain and the decrease of the family support domain. Overall, the increase of the age in the evaluation, better hearing and language skills, the mother's level of schooling and the family receptiveness correlated with the quality of life of children with cochlear implants.

**Conclusion:**

The influencing factors that correlated with the quality of life of the implanted children were the child's older age at the evaluation, the better hearing and language skills, the mother's level of schooling and the family receptiveness.

## Resumo

**Introdução:**

O impacto multidimensional da deficiência auditiva nas várias demandas da vida das crianças usuárias de implante coclear é representado por variáveis que podem influenciar os resultados de audição, de linguagem e de qualidade de vida desta população.

**Objetivo:**

Avaliar os fatores influenciadores na qualidade de vida de crianças com implante coclear, considerando a idade na cirurgia, a idade auditiva, a idade na avaliação, as habilidades auditivas, a linguagem falada, o grau de permeabilidade familiar, a escolaridade e o nível socioeconômico dos pais.

**Método:**

Participaram 30 crianças usuárias de implante coclear com idades entre 6 a 12 anos e seus respectivos pais. As crianças foram avaliadas pelos instrumentos *categories auditory performance*, pela categoria de linguagem e pelo questionário crianças com implante coclear: perspectivas dos pais. Os pais foram avaliados pela escala de envolvimento familiar.

**Resultados:**

O implante coclear impactou a qualidade de vida das crianças, com resultados mais significativos sobre o aumento do domínio relações sociais e a diminuição do domínio suporte da família. De maneira geral, maior idade na avaliação, as melhores habilidades auditivas e de linguagem, a escolaridade da mãe e a permeabilidade da família se correlacionaram com a qualidade de vida de crianças com implante coclear.

**Conclusão:**

Os fatores influenciadores que se correlacionaram com a qualidade de vida das crianças implantadas foram a maior idade na avaliação, as melhores habilidades auditivas e de linguagem, a escolaridade da mãe e a permeabilidade da família.

## Introduction

The benefits of a Cochlear Implant (CI) in children are well known. The success related to spoken language recognition and comprehension skills are well-established in both national and international literature.^1^, ^2^, ^3^, ^4^ However, a smaller number of studies is focused on the investigation of the quality of life of children with CI in common situations of daily life, such as: communication functionality, interactions with the social environment at home and at school, as well as their needs and desires. Few studies are directed at the influence of the several variables that involve the complex and multidimensional implantation process.^5^, ^6^, ^7^, ^8^, ^9^, ^10^, ^11^, ^12^

The use of specific tools to assess the quality of life of children with CI allows the assessment of the impact of hearing loss and the device use in everyday situations, beyond the evaluation of the hearing and spoken language skills provided by the formal clinical measures. The information acquired through the opinion of the parents, or in some situations, by the patients themselves, has the potential value of guiding conducts during the intervention process.^7^, ^9^, ^13^, ^14^

Thus, greater investments in the investigation of these other life demands of the child with a CI are necessary and may result in greater benefit, in addition to guiding the speech therapy, family counseling and guidance during the therapeutic process with a more individualized approach for each child.

Therefore, the aim of this study was to evaluate the factors influencing the quality of life of children with cochlear implants, considering the age at surgery, hearing age, age at evaluation, hearing skills, spoken language, degree of family receptiveness, the parents’ level of schooling and socioeconomic level.

## Methods

This was a cross-sectional, descriptive, quantitative study, approved by the Research Ethics Committee of Hospital de Reabilitação de Anomalias Craniofaciais of Universidade de São Paulo (HRAC/USP), Retirar under CAAE no. 61753416.0.0000.5441.

### Sample

Thirty children of both genders, aged between 6 years and 12 years, who were CI users enrolled in the Cochlear Implant Section (SIC) of HRAC/USP, and their respective parents participated in the study. The participants signed the Free and Informed Consent Form (FICF) and Assent Form. The sample consisted of a convenience sample, with parents and children attending the CI follow-up routine between March and August 2017.

The inclusion criteria of this study were: having a minimum of six complete years of age (72 months) to 12 incomplete years (144 months), having severe and/or profound bilateral sensorineural hearing loss, having undergone CI surgery according to the multifactorial criteria for cochlear implant surgery indication proposed by the interdisciplinary team of the Cochlear Implant Section (SIC-HRAC/USP), with total electrode insertion and during the sensitive period of auditory neural plasticity, up to three years and six months of age,^15^ having all electrodes active at the time of the evaluation, without prolonged interruption of the device use (>three months) in the last 12 months and undergoing speech therapy or having been discharged by the professional. Children with bilateral implants, Auditory Neuropathy Spectrum Disorder (ANSD), those with auditory nerve hypoplasia, with outer, middle or inner ear malformation, those with other loss associated with hearing impairment, and children who did not understand the instructions of the procedures proposed in the research were excluded. The demographic data on the children and their families are described in Table 1.

**Table 1:** Demographic characteristics of the studied variables regarding age at surgery, auditory age and age at the children's evaluation, socioeconomic classification and level of schooling of parents/guardians (*n* = 30).

Description	*M*	SD	Min	Max	1^st^ Quartile	Median	3^rd^ Quartile
Age at surgery (months)	22.9	7.9	9.0	37.0	17.0	21.5	29.2
Auditory age (months)	88.9	21.8	49.0	126.0	65.5	93.0	105.7
Age at evaluation (months)	114.3	19.5	76.0	143.2	106.0	116.5	126.5

Description	Classification	*n*	%
Etiology	Congenital	21	70
	Meningitis	3	10
	ICU	2	6.7
	Family history	2	6.7
	Progressive	1	3.3
	Icteric	1	3.3

Socioeconomic status	Lower-low	2	6.7
	Upper-low	11	36.7
	Middle-low	13	43.3
	Middle	1	3.3
	Upper middle	3	10.0

Level of schooling (father)	Incomplete Elementary School	4	14.3
	Complete Elementary School	2	7.1
	Complete High School	6	21.4
	Incomplete College/University	4	14.3
	Complete College/University	12	42.9

Level of schooling (mother)	Incomplete Elementary School	1	3.3
	Complete Elementary School	1	3.3
	Incomplete High School	2	6.7
	Complete High School	11	36.7
	Incomplete College/University	1	3.3
	Complete College/University	14	46.7

*M*, mean; SD, standard deviation; Min, minimum; Max, maximum.

### Procedures

The children's auditory performance was determined based on the Categories of Auditory Performance (CAP) scale^16^ (Table 2). The scores in the Disyllabic Words List test were used for the classification in these categories,^17^ as this was the most advanced test that the entire group was able to perform together at the last CI follow-up visit, as well as the Hearing Categories classification.^18^ The data of language considered was the Categories of Language classification.^19^ (Table 3). This information was collected from the standardized and validated medical records that were used as research material.

**Table 2:** Variable studied regarding the distribution of children according to CAP (*n* = 30).

	Categories of Auditory Performance (CAP)						
	1	2	3	4	5	6	7
*n*	–	–	–	5	4	3	18
%	–	–	–	16.7	13.3	10.0	60.0

**Table 3:** Variable studied regarding the distribution of children according to the Language Category (*n* = 30).

	Language Category				
	1	2	3	4	5
*n*	–	1	2	8	19
%	–	3.3	6.7	26.7	63.3

The Family Involvement Rating scale,^20^ translated into Brazilian Portuguese as “Escala de Envolvimento Familiar”,^21^ was applied by two researchers without previous contact with the participants to investigate the degree of family receptiveness in the therapeutic process. The results of this classification are shown in Table 4.

**Table 4:** Variable studied regarding the distribution of parents according to family receptiveness (*n* = 30).

Degree of family receptiveness	Below average	Average	Above average
*n*	3	13	14
%	10.0	43.3	46.7

The children's quality of life assessment was measured using the Children with Cochlear Implants: Parent's Perspectives (CCIPP) questionnaire,^22^, ^23^ translated and adapted into Brazilian Portuguese with the title “Crianças com Implante Coclear: Perspectivas dos Pais”.^7^ The CCIPP questionnaire was delivered to the parents with the appropriate instructions for filling it out, and without the researcher's aid or interference. The quantitative answers were analyzed using the software “Parent Questionnaire Manager – Parent Views and Experiences Questionnaire Data Entry” (ParQ120.exe., version 1.02: ISVR Software, Copyright 2003), prepared by the Ear Foundation team and available for download at http://resource.isvr.soton.ac.uk/audiology/Software/ParQ120.htm.

The more positive the answer, the greater the association between quality of life and the CI use from the parents’ perspective.

The statistical analyses were carried out using the software SPSS, version 18. Percentages, mean, standard deviation, minimum, maximum, 1^st^ quartile, median and 3^rd^ quartile values were used for the representation of variables: age at surgery, hearing age, age at evaluation, auditory skills, spoken language skills, degree of family receptiveness, level of schooling, and socioeconomic level of the parents. The inferential correlation analysis between the influencing variables and quality of life was performed using the non-parametric statistical dependence test between two variables, Spearman's rho Correlation Test. The level of significance was set at *p* ≤ 0.05%.

## Results

**Figure 1:**
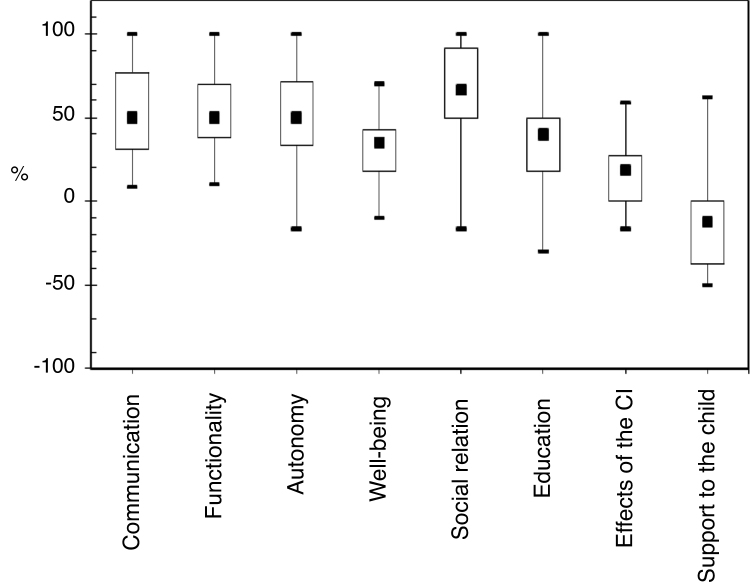
Median, 1^st^ quartile, 3^rd^ quartile, minimum and maximum values of the parents’ perceptions regarding the quantitative domains of the CCIPP questionnaire represented by box plots (*n* = 30).

Fig. 1 shows the medians of the parents’ answers to the CCIPP questionnaire in each of the evaluated quantitative domains. Statistically significant correlations between the CCIPP subscales are shown in Table 5. Table 6 shows the correlations between the CCIPP subscales and the study variables.

**Table 5:** Correlation between the quantitative domains of the CCIPP questionnaire (*n* = 30).

	Communication	Functionality	Autonomy	Well-being	Social	Education	Effects of CI
*Functionality*							
Rho	0.592^b^						
*p*	0.001						

*Autonomy*							
Rho	0.623^b^	0.452^a^					
*p*	0.000	0.012					

*Well-being*							
Rho	0.104	0.346	0.053				
*p*	0.586	0.061	0.782				

*Social*							
Rho	0.404^a^	0.075	0.466^b^	0.229			
*p*	0.027	0.692	0.009	0.224			

*Education*							
Rho	0.471^b^	0.604^b^	0.594^b^	0.013	0.194		
*p*	0.009	0.000	0.001	0.945	0.305		

*Effects of CI*							
Rho	0.207	0.132	0.436^a^	0.085	0.271	0.416^a^	
*p*	0.272	0.486	0.016	0.657	0.148	0.022	

*Support*							
Rho	−0.109	0.173	−0.092	0.400^a^	−0.095	0.021	−0.132
*p*	0.568	0.361	0.630	0.028	0.616	0.911	0.485

CI, Cochlear Implant; CCIPP, Children with Cochlear Implants: Parent's Perspectives.

a Significant values = *p* ≤ 0.05.

b Spearman's correlation coefficient test (*p* ≤ 0.01).

**Table 6:** Correlation between the quantitative domains of the CCIPP questionnaire and the variables: age at surgery, auditory age, and age at evaluation, CAP, language category, family receptiveness, parents’ level of schooling and socioeconomic levels (*n* = 30).

Domains	Age at surgery	Auditory age	Age at evaluation	CAP	Language category	Family receptiveness	Paternal level of schooling	Maternal level of schooling	Socioeconomic level
*Communication*									
Rho	−0.113	0.034	−0.032	0.464^b^	0.366^a^	0.426^a^	0.222	0.640^b^	0.259
*p*	0.552	0.860	0.866	0.010	0.047	0.019	0.257	0.000	0.166

*Functionality*									
Rho	−0.182	0.153	0.108	0.284	0.117	0.052	0.135	0.196	−0.055
*p*	0.335	0.421	0.570	0.129	0.539	0.785	0.494	0.300	0.774

*Autonomy*									
Rho	−0.134	0.319	0.320	0.266	0.437^a^	0.319	0.063	0.253	0.029
*p*	0.479	0.086	0.084	0.156	0.016	0.086	0.749	0.178	0.880

*Well-being*									
Rho	−0.139	−0.194	−0.274	0.142	−0.064	−0.045	0.167	0.045	−0.147
*p*	0.465	0.303	0.142	0.455	0.736	0.815	0.396	0.813	0.437

*Social*									
Rho	−0.010	−0.052	−0.049	0.036	0.260	0.199	0.201	0.460^a^	0.158
*p*	0.959	0.786	0.797	0.848	0.165	0.291	0.305	0.010	0.404

*Education*									
Rho	−0.073	0.332	0.417^a^	0.442^a^	0.329	0.477^b^	0.087	0.280	0.114
*p*	0.703	0.073	0.022	0.014	0.076	0.008	0.659	0.134	0.550

*Effects of CI*									
Rho	−0.204	0.113	0.027	0.235	0.299	0.185	−0.227	0.039	−0.141
*p*	0.280	0.550	0.889	0.210	0.108	0.328	0.245	0.837	0.458

*Support*									
Rho	0.030	−0.169	−0.121	0.108	0.035	0.000	0.030	−0.026	−0.158
*p*	0.873	0.371	0.526	0.571	0.855	0.999	0.880	0.891	0.404

CI, Cochlear Implant.

a Significant values = *p* ≤ 0.05.

b Spearman's correlation coefficient test (*p* ≤ 0.01).

## Discussion

Quality of life assessment involves several factors, such as physical and emotional well-being, self-esteem, family, friends, school, satisfaction with the CI, social aspects, mobility, self-care, pain, telephone use, speech comprehension, environmental sound perception, communication, self-sufficiency, use of electronic devices, other peoples’ attitudes, self-confidence, preferences, interests and ethical and moral values.^7^, ^8^

In this sense, measuring the quality of life in the pediatric population requires the use of sensitive measures to assess these aspects. The use of specific evaluation tools is highly advantageous, because it allows the assessment of parents’ perceptions and provides information about auditory and speech language development and expresses significant changes in the perception of the family regarding the CI user's satisfaction. These data help professionals in the intervention process.^7^, ^8^, ^24^

In the last 10 years, four studies^7^, ^9^, ^14^, ^25^ in Brazil used the specific CCIPP questionnaire to evaluate the quality of life of children with CI. Some authors^14^, ^25^ identified significant gains in the quality of life of children and their families after the use of CI in all subscales of CCIPP, with more satisfactory expectations of parents in the domains of social relations, followed by autonomy, while others^7^, ^9^ found greater emphasis on the domains of autonomy, followed by social relations. International studies have also confirmed that CI positively interferes with the quality of life of its users through improvement in the domains of social relations, autonomy and communication. According to the authors, these are the first benefits to be reported after the use of the CI.^13^, ^22^, ^26^, ^27^, ^28^, ^29^, ^30^, ^31^

The results of the present study are in agreement with previous findings. From the parents’ perspective, the CI improved the quality of life in all domains related to the child and in one of the domains related to the family. The subscale of social relations showed a more significant impact, followed by communication, functionality, autonomy, education, well-being and happiness, of the effects of CI on the family, and of the support and assistance given to the child (Fig. 1).The fact that the highest quality of life indexes associated with the CI are recorded in these domains suggests that for parents, hearing capacity and language acquisition are applied in a practical way on the daily life of these children and contribute to the inclusion and increase of social activities (family and non-family) as a result of the acceptance of their capabilities in complex situations of communication exchanges. Furthermore, the use of the device and the acquisition of communication skills make the children more independent individuals socially and emotionally.^9^, ^14^, ^32^

Among the evaluated domains, the subscale support and assistance to the child recorded the lowest indexes of quality of life scores in relation to the other scales. The same was observed in other studies^7^, ^9^, ^14^ and although it seems to be a negative result, it suggests that children with good quality of life indexes are less dependent on parental support and assistance, making it a positive finding. Decreased parental support results in increased autonomy, citizenship and the projection of these children as future students and independent professionals.^7^, ^9^, ^13^, ^14^

In the analysis of the CCIPP subscales (Table 5), autonomy and communication were directly correlated with the highest number of domains. In some studies^7^, ^9^, ^13^, ^14^, ^25^ the domains of communication, well-being and happiness and overall functioning showed the highest number of correlations with the other subscales of the questionnaire. These associations suggest that the acquisition and development of spoken language are associated with the development of other skills, which give the children independence and increase the percentage of quality of life, considered as positive effects of the implantation from the parents’ point of view.

Even with overall good quality of life results, the minimum and maximum response values and the standard deviation between the subscales showed the variability of results, distancing some children from the group's mean values, due to the different perspectives shown by some parents (Fig. 1). Researchers who obtained similar results^9^ indicate that the justification for the group's discrepancy of mean values can be considered based on the performance of each child measured by the speech perception and spoken language tests, as well as by the individual perception, expectations, insecurities and anxieties of each family in relation to the overall development of these children.^9^, ^33^, ^34^, ^35^, ^36^

For children who did not reach the highest percentages of quality of life after the CI use (Fig. 1), the importance of identifying the variables with the greatest influence on these results is highlighted, aiming at increasing the investments so that the patients and their families can perceive and acquire all the benefits provided by the CI.^3^, ^33^, ^36^, ^37^, ^38^, ^39^, ^40^ Considering this, the present study investigated the variables influencing the quality of life of children with cochlear implants (Table 6). Even though some authors^8^, ^15^, ^41^ have suggested that the children's best developmental results and the quality of life are associated with the performance of the CI surgery in the first years of life, due to the capacity to reorganize the neuronal plasticity and the adequate maturation of the Central Auditory Nervous System as well as the time of CI use,^6^, ^8^, ^9^ in this study, the age at surgery and hearing age did not show a statistically significant impact on the quality of life. However, results such as these are not uncommon and agree with similar investigations regarding age at implantation^6^, ^10^ and the time of device use,^10^ both with no significant correlations. On the other hand, the increase of the age at the evaluation showed a positive impact on the education domain. The influence of this aspect on the quality of life was previously studied by authors^6^, ^10^ who did not find statistically significant correlations. Another study,^8^ in turn, identified that chronologically older children had statistically significant positive correlations relative to quality of life.

Table 6 also demonstrates that children with higher categories in CAP showed influence on the domains of communication and education in the CCIPP, whereas language skills were related to the domains of communication and autonomy of the studied group. The correlation between hearing and spoken language skills and quality of life has been studied by several authors with variable results. Some authors^5^, ^6^, ^7^, ^8^, ^12^, ^25^ identified significant correlations between auditory performance and quality of life, while other evidence^6^, ^9^, ^11^ did not find any statistically significant correlations. Language studies^7^, ^8^, ^25^ showed positive correlations with quality of life, unlike other authors, who did not observe the impact of this variable.^6^

Considering the multidimensionality and multifactorial aspects of the implantation and development process of children with cochlear implants that occur at different temporal scales, it is natural that in some studies, certain variables have a strong effect on one group of children but not on others, explaining the variability of results found in this and other studies.

Because of this, variables related to the family nucleus such as the socioeconomic level, parental level schooling and family receptiveness in speech therapy are also receiving attention from the literature due to the fact that small children usually spend most of their time interacting with their parents and depend on them for the full rehabilitation process. In this study, the mother's level of schooling was positively correlated with the domain of communication and social domain, and more receptive parents had an impact on the child's communication and education. No statistically significant correlations were found between quality of life and socioeconomic aspects (Table 6). In the literature, few studies have found a correlation between these variables.^6^ It is encouraging to see that the parents’ commitment to the therapeutic process is free from the influence of their purchasing power and/or educational level.

Overall, the quality of life was influenced by five of the investigated variables. When analyzed individually, family receptiveness appeared as one of the most significant variables, acting as a strong predictive marker of development and better quality of life in these children. Considering the different variables that involve the development of children with CI, it is evident the complexity that includes the control of all influencing factors involved in the CI indication, adaptation and monitoring of this population. The desirable balance between the investigated variables alone does not guarantee the full performance of auditory skills, language skills and quality of life.

## Conclusion

The influencing factors that correlated with the quality of life of the children with CI were the older age at the evaluation, the better hearing and language skills, the mother's level of schooling and the family's receptiveness.

This knowledge can guide the speech therapist in charge of the rehabilitation to promote improvements in the planning of specialized speech therapy, consistent with the individuality of the child and his/her family, contemplating personalized guidelines aimed at different socioeconomic and instructional realities, guaranteeing to the families the same possibilities of access to information and opportunities for development. Moreover, this evidence can help the interdisciplinary teams at the stage of cochlear implant indication and when evaluating the progress of the results, guiding the postoperative follow-up based on the specific characteristics of each child and family regarding these factors.

## Conflicts of interest

The authors declare no conflicts of interest.
